# Biomarker RIPK3 Is Silenced by Hypermethylation in Melanoma and Epigenetic Editing Reestablishes Its Tumor Suppressor Function

**DOI:** 10.3390/genes15020175

**Published:** 2024-01-28

**Authors:** Sarah Arroyo Villora, Paula Castellanos Silva, Tamara Zenz, Ji Sun Kwon, Nico Schlaudraff, Dafina Nitaj, Cornelia Meckbach, Reinhard Dammann, Antje M. Richter

**Affiliations:** 1 Institute for Genetics, Justus-Liebig-University Giessen, 35390 Giessen, Germany; 2 Department of Mathematics, Natural Sciences and Computer Science, University of Applied Sciences Mittelhessen, 35390 Giessen, Germany

**Keywords:** RIPK3, DNA (hyper)methylation, skin cutaneous melanoma, epigenetic editing, CRISPR-Cas9, deactivated dCas9, tumor suppressor gene, biomarker, cancer

## Abstract

For several decades, cancers have demonstrably been one of the most frequent causes of death worldwide. In addition to genetic causes, cancer can also be caused by epigenetic gene modifications. Frequently, tumor suppressor genes are epigenetically inactivated due to hypermethylation of their CpG islands, actively contributing to tumorigenesis. Since CpG islands are usually localized near promoters, hypermethylation of the promoter can have a major impact on gene expression. In this study, the potential tumor suppressor gene Receptor Interacting Serine/Threonine Protein Kinase 3 (RIPK3) was examined for an epigenetic regulation and its gene inactivation in melanomas. A hypermethylation of the *RIPK3* CpG island was detected by bisulfite pyrosequencing and was accompanied by a correlated loss of its expression. In addition, an increasing *RIPK3* methylation rate was observed with increasing tumor stage of melanomas. For further epigenetic characterization of RIPK3, epigenetic modulation was performed using a modified CRISPR/dCas9 (CRISPRa activation) system targeting its DNA hypermethylation. We observed a reduced fitness of melanoma cells by (re-)expression and demethylation of the *RIPK3* gene using the epigenetic editing-based method. The tumor suppressive function of RIPK3 was evident by phenotypic determination using fluorescence microscopy, flow cytometry and wound healing assay. Our data highlight the function of RIPK3 as an epigenetically regulated tumor suppressor in melanoma, allowing it to be classified as a biomarker.

## 1. Introduction

Skin cancer is one of the most aggressive cancers worldwide and can be divided into different types such as malignant melanoma, squamous cell carcinoma (SCC) and basal cell carcinoma (BCC). Malignant melanoma is a form of skin cancer that develops from the pigment-forming cells, the melanocytes. Malignant melanoma accounts for only a small number of all skin cancer cases but is responsible for a large proportion of deaths from skin cancer [1]. In addition to genetic causes, cancer can also be caused by epigenetic modifications such as DNA methylation. DNA methylation is caused by DNA methyltransferases (DNMTs), and the addition of a methyl group at the C-5 position of a cytosine occurs in a CpG context [2,3]. The result is the formation of a 5-methylcytosine (5mC) that functions as a silencing signal for a closed state of the corresponding promoter. More than half of all genes in the vertebrate genome have CpG-containing regions (over 55%), the so-called CpG islands, while the rest of the genome contains less than one percent of CpGs [4]. CpG islands are approximately 1 kilo base (kb) in length, often located in promoter regions and, therefore, in the vicinity of transcriptional start sites (TSSs) [5,6]. DNA methylation in these promoter regions can cause a change in the transcription and expression of a gene by the formation of gene silencing complexes that contain, e.g., methyl-binding proteins, histone deacetylases and transcriptional repressors [7]. In cancer, tumor suppressor genes are often epigenetically inactivated due to hypermethylation of their CpG islands. We have previously identified several epigenetically silenced tumor suppressor genes in various tumor entities [8,9,10].

One of the characteristic abilities of malignant tumors is the evasion of cell death. Programmed cell death is regulated by evolutionarily conserved signaling pathways [11,12,13]. The best-known pathways of programmed cell death are apoptosis and necroptosis, with necroptosis having inflammatory characteristics [14]. RIPK3 plays a key role in necroptotic signaling and is, therefore, of major importance in the study of tumorigenesis. The approximately 60 kDa Receptor Interacting Serine/Threonine Protein Kinase 3 (RIPK3) is encoded by the *RIPK3* gene, which is located on chromosome 14 in the q12 region [NCBI, UNIPROT]. *RIPK3* contains 10 exons and harbors an annotated CpG island (357 base pairs (bp)), located downstream of its promoter [UCSC]. With its RIP homotypic interaction motif (RHIM) domain, RIPK3 is able to bind RIPK1 and can form the necrosome [15]. The necrosome recruits and phosphorylates the mixed lineage kinase domain-like protein (MLKL), which oligomerizes and induces necroptosis at the cell membrane [15]. In some tumor entities, the gene is already considered a potential tumor suppressor gene and exhibits aberrant expression [16,17,18,19]. Geserick et al. previously showed the loss of RIPK3 expression in malignant melanoma and the resulting necroptosis resistance [20]. However, from their study, it remained unclear how the RIPK3 inactivation is occurring. Our data now explain the loss of expression of RIPK3 in malignant melanoma, by highlighting that RIPK3 is an epigenetically silenced tumor suppressor. In addition, reestablishing endogenous *RIPK3* expression resulted in reestablishing its tumor-suppressive function.

## 2. Materials and Methods

**Analysis of publicly accessed data and origin of data.** Gene expression, promoter methylation, ChipSeq analysis and Kaplan–Meier calculations were performed using the R2 Genomics Analysis and Visualization Platform [21]. For methylation profiling, we used Wanderer [22]. For gene expression in melanoma, we used Gepia (http://gepia.cancer-pku.cn (accessed on 24 November 2023)). For correlation of the methylation and expression, we used Shiny Methylation Analysis Resource Tool (http://www.bioinfo-zs.com/smartapp/ (accessed on 24 November 2023)). For mutational analysis of *RIPK3*, we used cBioPortal for Cancer Genomics (https://www.cbioportal.org (accessed on 24 November 2023)).

**Statistical analysis.** Microsoft Excel was utilized for statistical analysis. A one-tailed Welch’s *t*-test or Fisher exact test was applied to distinguish between two samples. The Fisher exact test involved categorizing data for each sample into two groups, with the specific criteria for this division detailed at the relevant positions in the manuscript. Epigenetic editing: Transfection, followed by all analyses, was performed in biological triplicate. Quantitative expression analysis: *GAPDH* in technical triplicate, *RIPK3* and *DNMT3A* in technical quadruplicate. Methylation analyses and Western blot in technical triplicates. Cell cycle analysis by flow cytometry: RIPK3 overexpression in HEK293T (investigated number (n)) n = 10,769, in MeWo n = 32,759. Localization/morphology studies using Fluorescence microscopy: RIPK3 overexpression HEK n = 344, C918 n = 327, IGR1 n = 346, MeWo n = 420, SkMel13 n = 375. RIPK3 epigenetic editing in MeWo: VP160-dCas9 n = 126, TET1-dCas9 n = 141.

**Methylation analysis.** DNA was isolated by phenol-chloroform extraction. Promoter methylation was analyzed by combined bisulfite restriction analysis (COBRA) and bisulfite pyrosequencing as described previously [23,24]. For bisulfite treatment, we used EZ DNA Methylation Kit (Zymo Research D5001, Zymo Research Europe GmbH, Freiburg, Germany). For CoBRA and Pyrosequencing, bisulfite-treated DNA was PCR amplified (with 0.2 mM dNTP mix, 1.5 mM MgCl_2_, 10 pmol of each primer, 1.5 U Taq polymerase, 50 cycles). Methylation quantification was performed by PyroMark Q24 (QIAGEN GmbH, Hilden, Germany). Three CpGs are included in the *RIPK3* analyzed region, and mean methylation was calculated. In vitro methylation of genomic DNA was carried out using the CpG methyltransferase *M.Sss*I, based on the methyltransferase gene from *Spiroplasma sp.* strain MQ1 (NEB M0226S). An in vitro methylated DNA (pos.) served as the methylation positive control, while DNA extracted from *buccal mucosa* (neg.) served as the methylation negative control. *RIPK3* COBRA PCR-Primers (5′-TTTAGAGGTTTTTTGGATATTTTTTAGTT-3, 5′-ACACAATAACTCCACCTTTTAACCAA-3′, 5′-TTCTCTAAACRAATCTATAAAACTCTCTAAA-3′) were used to analyze promoter methylation.

**RNA expression analysis.** RNA was isolated from human cell culture using Isol-RNA lysis procedure (Trizol, Thermo Fisher Scientific, Dreieich, Germany). RNA was *DNase*I (Thermo Fisher Scientific, Dreieich, Germany) treated and reverse transcribed by MMLV (Moloney Murine Leukemia Virus, Promega Corporation, Mannheim, Germany). Quantitative RT–PCR was performed with SYBR select (Thermo Fisher Scientific, Dreieich, Germany) using Rotor-Gene 3000 (QIAGEN GmbH, Hilden, Germany) and normalized to *GAPDH*. *RIPK3* Real-Time Primer (5′-CGTCAAGTTATGGCCCAGCG-3′, 5′-CCGTGGATATCGCCTTCGAG-3′); *GAPDH* Real-Time Primer (5′-TGGAGAAGGCTGGGGCTCAT-3′, 5′-GACCTTGGCCAGGGGTGCTA-3′) and *DNMT3A* Real-Time Primer (5′-ATGTGGTGGCCATGGGCGTTAGTG-3′, 5′- TCCTTTCGAGCTCAGTGCACCACA-3′) were used to analyze expression.

**Epigenetic editing/ Epigenetic therapy by CRISPR-dCas9.** The pioneering work of the CRISPR-Cas technique was successfully applied to mammalian cells and has now been developed into a versatile and widely studied tool in genetics [25,26,27]. We are using the nuclease deficient dCas9 with point mutations of its two nuclease domains HNH/RuvC, rendering it unable to cleave DNA but retaining the ability to target genomic regions [25,28]. We are using fusion proteins of the dCas9 enzyme to effector domains/gene-regulatory proteins, which enables stable and efficient transcriptional activation, with the site of delivery determined solely by a co-expressed short guide (sg)RNA [29]. We used the VP160 transcription activator, originally from herpes virus protein VP16, and P300 (histone acetyltransferase, catalytic domain, K3K27ac) [30,31,32]. In addition, TET1 is the ten-eleven translocation methylcytosine dioxygenase 1, and its catalytic domain is used for CRISPR approaches [33]. As a control, we used dCas alone, without effector, that was reported to induce moderate demethylation that was replication-dependent [34]. For epigenetic editing, the cells were transfected in 6-well dishes at an initial density of approximately 80% with a total of 4 µg plasmid DNA. For transfection, X-tremeGENE HP (Roche Diagnostics, Rotkreuz, Switzerland) or polyethylenimine (Sigma-Aldrich Chemie GmbH, Steinheim, Germany) was used in combination with serum-reduced medium Opti-MEM (Gibco: Thermo Fisher Scientific, Dreieich, Germany), according to the manufacturer’s protocol. A 6-well dish of cells with transfected EYFP served as a transfection control. The medium was changed 5 h after transfection. Transfection efficiency was checked after 24 h by fluorescence microscopy. Puromycin (Gibco: Thermo Fisher Scientific, Dreieich, Germany ) selection was performed at 10 µg/mL (HEK293T) or 2.5 µg/mL (MeWo) from 24 h after transfection. RNA and DNA were isolated 96 h after transfection. *RIPK3* sgRNAs/Oligos were positioned/generated using Benchling and cloned into modified px459 delSpCas9(BB)-2A-Puro V2.0 (modified Addgene #62988, delCas9, available on request) […deutschm.]. *RIPK3* sgRNAs are #1, #2, #3, #4, #5, #6 and are positioned relative to TSS at -246 #1, -147 #2, + 36 #3, +241 #4, +434 #5 and +482 #6. A combination of all sgRNAs was used for all experiments. As the non-guided control, we used a sgRNA without a human target sequence alignment.

**Cell culture, cell cycle analysis and localization/morphology studies.** Cell lines were grown in appropriate medium (DMEM) supplemented with 10% FCS and 1% Penicillin/Streptomycin under cell culture conditions (37 °C, 5% CO_2_). Cell lines were transfected using X-tremeGENE HP (Roche, Diagnostics, Rotkreuz, Switzerland) or Polyethylenimin (Sigma-Aldrich Chemie GmbH, Steinheim, Germany) with either 4 µg (6 well) or 10 µg (10 cm dishes). Regarding flow cytometry analysis, cells were transfected and ethanol-fixed at indicated time points. The following day, cells were treated with 50 µg/mL RNaseA for 30 min at 37 °C. Subsequently, cells were stained with 50 µg/mL propidium iodide prior to measuring DNA content in FACSCantoII (BD Biosciences). BD FACSDiva™ Software v. 6.1.3 (BD Biosciences, Heidelberg, Germany) was used for measurement/gating to distinguish transfected fluorescent cells and to determine cells in G0/G1, S and G2/M phase of the cell cycle [35]. For localization analysis, cells were seeded on glass slides and transfected the following day. Cells were fixed with 3.7% formaldehyde at corresponding time points, permeabilized using tritonX, stained with DAPI (0.1 µg/mL in PBS, Sigma-Aldrich Chemie GmbH, Steinheim, Germany), embedded in anti-fading with Mowiol (Sigma-Aldrich Chemie GmbH, Steinheim, Germany ) and analyzed with Axio Observer Z1 (Carl Zeiss AG, Oberkochen, Germany) under 63x magnification and ZEISS ZEN 3.7 software (Carl Zeiss AG, Oberkochen, Germany). We generated a kinase dead domain for RIPK3 (RIPK3 KD) in which the ATP binding pocket of the RIPK3 kinase domain (K50A) was mutated by site-directed mutagenesis (QuikChange Lightning Kit, Agilent Technologies, Santa Clara, CA, USA). Subsequently, RIPK3 KD served as control. Cellular morphology was assessed by the category of nuclei shape and nuclei size. The measurement of cell nucleus size was performed manually and always in the same way (horizontally) (Figure S7). For Aza-2′-deoxycytidine (Aza) treatment, cells were split to 10% density, and Aza was added with fresh medium on four consecutive days at a working concentration of 5 µM or mock treated before cell isolation. For wound healing assay, cells were transfected as described and grown to confluency before wound/scratch was placed with pipette tip. Wound closure was measured at day 0 and 24 h later and is depicted in % gap closure, assuming that time 0 corresponds to a wound width of 100%.

**Western blotting.** Proteins were separated via SDS-PAGE and Western blotted onto PVDF membrane (Merck-Immobilon, Darmstadt, Germany) for antibody-based detection. Luminata Crescendo Western HRP substrate (Merck-Millipore, Darmstadt, Germany) was used for detection with a VersaDoc Imaging System (BioRad Laboratories Inc., Hercules, CA, USA). The following antibodies were used: a-Vinculin V9131 mAb mouse of Sigma, a-RIP3 (a-RIPK3) (#10188, Cell Signaling Technology, Danvers, TX, USA), HRP-coupled secondary antibodies anti-rabbit IgG-HRP (sc-2357, Santa Cruz Biotechnology, Dallas, TX, USA) and anti-mouse IgGκ BP-HRP (sc-516102, Santa Cruz Biotechnology, Dallas, TX, USA).

**Tissue and cell lines.** The kidney cell line HEK293T (HEK, RRID: CVCL_0063) and melanoma cell lines C918 (RRID: CVCL_8471), IGR1 (RRID: CVCL_1303), MeWo (RRID: CVCL_0445), SkMel13 (RRID: CVCL_6022) and SkMel19 (RRID: CVCL_6025) were used. All cell lines were mycoplasma-free and authenticated using short tandem repeat (STR) profiling within the last 3 years (Eurofins Genomics, Ebersberg, Germany). All 56 primary samples and 6 cell lines used are listed in the Supplementary Materials (Table S1). The study was conducted according to the Declaration of Helsinki Principles. All patients signed informed consent at initial clinical investigation. The study was approved by local ethics committees.

## 3. Results

### 3.1. Epigenetic Silencing of RIPK3 in Melanomas

**Figure 1:**
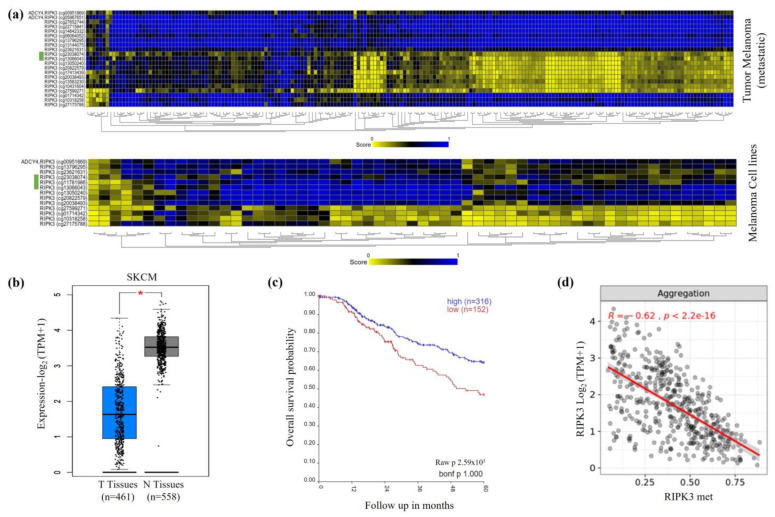
Increased *RIPK3* methylation and reduced *RIPK3* expression of melanomas. (**a**) *RIPK3* hypermethylation in primary melanoma and cell lines as methylation heatmaps via Illumina Methylation Assay-450k-Array (n = 196, Tumor melanoma (metastatic)-Jönssen-196-ilmnhmepic by R2; Cell line Cancer Pharmacogenomic-Esteller-1028-custom-ilmnhm450 by R2). The CpG probes cg23038074, cg11781986 and cg13066043 of CpG island 33 of *RIPK3* are marked in green. (**b**) Reduced *RIPK3* expression in melanoma vs. normal control tissue, significant (*p* < 0.01, TPM, log2, TCGA by Gepia). (**c**) Kaplan–Meier patient survival curve shows lower probability of survival with low *RIPK3* expression (n = 470, Tumor Skin Cutaneous Melanoma-TCGA-470-rsem-tcgars by R2). (**d**) Correlation of *RIPK3* expression and methylation in melanoma (within *RIPK3* CpG island). The mean value (aggregation) of the three annotated CpG probes is shown (n = 470; Methylation450k, Pan-Cancer Atlas; TOIL RSEM tpm UCSC Toil RNAseq Recompute (Gene expression RNAseq) by Shiny Methylation Analysis Resource Tool).

We analyzed the CpG island of *RIPK3* that is located downstream of its promoter (Figure S1) to assess the status of *RIPK3* hypermethylation in malignant melanomas. The CpG island region also contains three annotated CpG probes, cg23038074 (CpG1), cg11781986 (CpG2) and cg13066043 (CpG3) (Infinium HumanMethylation BeadChip, Illumina Methylation Assay-450k-Array), which were used to analyze the methylation status of *RIPK3* (Figure S1). Metadata already showed an increased occurrence of methylation of *RIPK3* in various tumor tissues and cancer cell lines, particularly within its CpG island (Figure S2). In contrast, *RIPK3* is rarely mutated in melanoma (Figure S3). Regarding DNA methylation of the *RIPK3* locus, the three annotated CpG probes were increasingly (significantly) methylated from normal tissues to primary tumors and to cancer cell lines (Figure S4). We observed heavy Enhancer of zeste homologue 2 (EZH2) coverage by ChipSeq (data mining, Figure S1), which further supports the concept of an epigenetic inactivation of RIPK3. EZH2 is part of the polycomb repressive complex 2 (PRC2) that leads to heterochromatin formation [36]. In addition to increased methylation, a loss of expression of *RIPK3* in cancer cell lines could be detected in further data sets (Figure S5). Methylation heatmaps generated with data from Illumina Methylation Assay-450k-Array showed an increased methylation rate of the CpG probes in the CpG island of RIPK3 in metastatic melanomas as well as in melanoma cell lines (Figure 1a) in comparison to normal tissues (Figure S2). *RIPK3* expression was strongly reduced in tumor samples of skin cancer (malignant melanoma, The Cancer Genome Atlas Program (TCGA)) in comparison to normal control skin samples (Figure 1b). Patient survival was also reduced with reduced *RIPK3* expression (Survival by Kaplan–Meier) (Figure 1c). Using the *RIPK3* CpG probes of its CpG island, low expression correlated with high *RIPK3* methylation (Figure 1d, Figure S6a), supporting the idea of an epigenetically inactivated tumor suppressor.

### 3.2. Epigenetic Editing by CRISPR-dCas9 (CRISPRa) System

**Figure 2:**
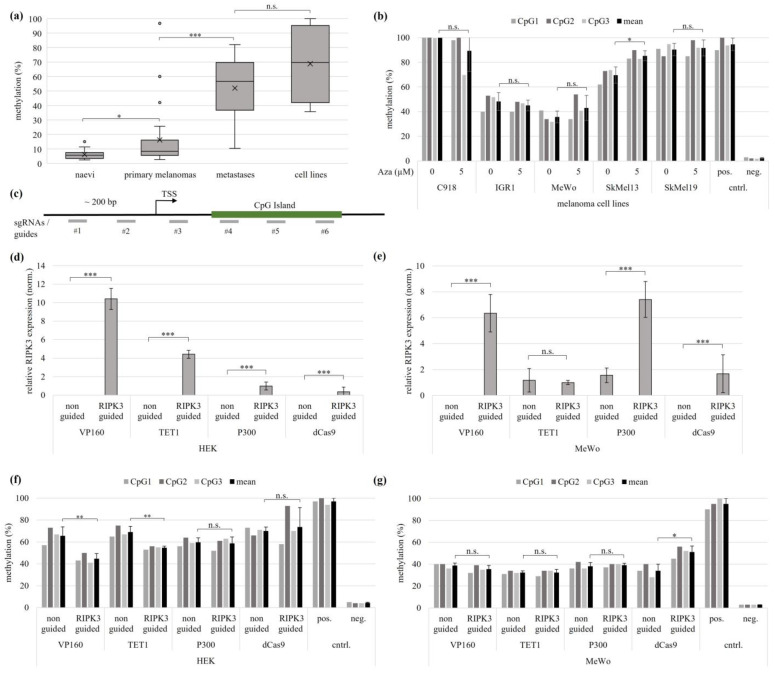
Epigenetic editing causes demethylation and re-expression of RIPK3 in melanoma and kidney cell lines. (**a**) Pyrosequencing shows rising *RIKP3* methylation level with increasing tumor stage of melanoma (naevi n = 13, primary melanomas n = 24, metastases n = 19, cell lines n = 5). (**b**) The five different Aza-treated melanoma cell lines showed no *RIPK3* demethylation by pyrosequencing. (**c**) Schematic representation of the guide target positions for the *RIPK3* promoter. (**d**,**e**) Quantitative expression analysis in MeWo and HEK shows a higher *RIPK3* re-expression with VP160-dCas9 than with TET1 dCas9. The values were normalized to *GAPDH*. (**f**) In the experiment corresponding to (**d**), the strongest *RIPK3* demethylation using VP160-dCas9 in HEK is observed (pyrosequencing). (**g**) In the experiment corresponding to (**e**), no demethylation by epigenetic editing in MeWo is observed (pyrosequencing). (**a**,**b**,**d**–**g**) *t*-test for statistical analysis, * *p* < 0.05, ** *p* < 0.01, *** *p* < 0.001, n.s. = not significant.

Based on the metadata, we investigated the epigenetic silencing of RIPK3 in melanomas. We performed quantitative methylation analysis by bisulfite pyrosequencing and quantitative expression analysis by RT-PCR. We analyzed patient samples and melanoma cancer cell lines. To investigate the methylation rate of the *RIPK3* CpG island with advancing tumor progression, we quantified *RIPK3* methylation by pyrosequencing of patient material of naevi, dysplastic naevi, primary melanomas, metastases, and melanoma cell lines. We observed low *RIPK3* methylation of naevi and increasing methylation for primary melanoma, metastases, and melanoma cell lines (Figure 2a). We did not observe differences between naevi and dysplastic naevi (Table S1). The examined naevi samples showed a mean methylation of 6%, the primary melanomas 16%, the metastases 50% and the cell lines 69% (Figure 2a, Table S1). The significant increase in the methylation rate with increasing tumor stage suggests an epigenetic regulation of RIPK3 in advanced tumors.

In order to induce pharmacological DNMT inhibition and DNA demethylation, the melanoma cell lines C918, IGR1, MeWo, SkMel13 and SkMel19 were treated for four consecutive days with 5 µM of the DNA-methyltransferase (DNMT)-inhibiting agent 5-aza2′deoxycytidine (vs. mock treatment 0 µM Aza). Pyrosequencing showed an average methylation of 90% and 100% for the untreated cells of SkMel19 and C918, while IGR1 showed a methylation of 48% and MeWo 36%. SkMel13 showed a methylation rate of 70%. The average methylation rate of the three CpG probes in the CpG island of *RIPK3* was measured. After treatment with Aza, no significant *RIPK3* demethylation was observed in C918, IGR1, MeWo, SkMel13 and SkMel19 (Figure 2b).

Due to the failure to achieve demethylation by pharmacological DNMT inhibition, we proceeded with targeted epigenetic editing for RIPK3 demethylation and RIPK3 expression analyses in the cell lines HEK and MeWo. Epigenetic editing is based on a modified CRISPR system using a deactivated Cas9 (dCas9) in combination with an effector protein and guidance of the system by target-specific RNA guides. For targeting epigenetic modifications, *RIPK3*-specific sgRNAs were co-transfected with the regulatory effector protein (dCas9 and effector). To analyze the regions upstream of the transcriptional start (TSS), between the TSS and the CpG island and a major part of the CpG island, the target sequences of the sgRNAs were selected for an approx. distance of about 200 bp (Figure 2c). The three effectors VP160, TET1 and P300 were used to examine epigenetic editing effects on RIPK3 demethylation and re-expression. A quantitative expression analysis of HEK cells showed RIPK3 re-expression for all effectors used, with VP160-dCas9 showing the strongest re-expression, followed by TET1-dCas9, P300-dCas9 and the dCas9 control. The re-expression of VP160-dCas9 was 2.4-fold stronger than the re-expression of TET1-dCas9 (Figure 2d). In the MeWo cells, however, only the effectors VP160-dCas9 and P300-dCas9 induced re-expression, followed by dCas9 alone. No re-expression could be shown by TET1-dCas9 (Figure 2e). In addition, and as an internal control, we confirmed that *DNMT3A* expression levels after epigenetic editing remained unaltered (Figure S6b). Quantitative methylation analysis showed that VP160-dCas9-transfected HEK cells achieved the greatest decrease in methylation, by 32%. The TET1-dCas9 induced a significant *RIPK3* demethylation of 21%. However, no significant decrease in methylation was observed for either P300-dCas9 or dCas9 alone (Figure 2f). Pyrosequencing of the MeWo cells showed no demethylation by all effectors used. However, when using dCas9 alone, a significant increase in methylation of RIPK3 was observed (Figure 2g). Our results show that VP160-dCas9 was able to cause re-expression in the melanoma cell line MeWo and kidney cell line HEK, whereas RIPK3 demethylation could only be observed in HEK.

### 3.3. Reactivation of the Tumor Suppressive Function of RIPK3 using Overexpression and Epigenetic Editing

**Figure 3:**
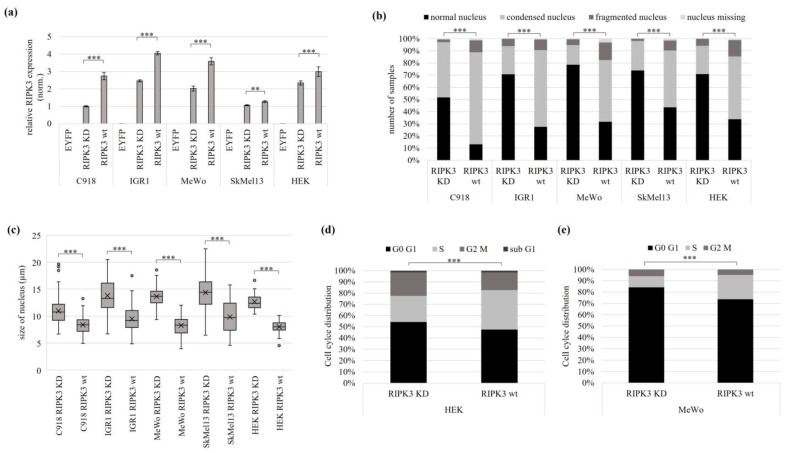
Tumor suppressive function of RIPK3 in melanoma and kidney cells. Different melanoma cell lines and HEK cells were transfected with EYFPC2-RIPK3 KD (kinase dead) and EYFPC2-RIPK3 wt (wild type). The EYFPC2 empty vector was used as a negative control. (**a**) Overexpression of RIPK3 KD and RIPK3 wt 24 h post transfection. The values were normalized to *GAPDH* and set to a value of one in relation to the cell line C918 with RIPK3 KD. (**b**) Melanoma and HEK cells with overexpressed RIPK3 wt show increased numbers of condensed and fragmented nuclei. The cells were fixed on glass slides 24 h after transfection and analyzed by fluorescence microscopy (64× objective). DAPI was used for nuclear staining (number of cells analyzed: C918 n = 327; IGR1 n = 346; MeWo n = 420; SkMel13 n = 375; HEK n = 344). (**c**) Reduced nucleus size after RIPK3 wt overexpression (equivalent experiment from (**b**)). The cell nucleus size was determined by fluorescence microscopy (C918 n = 172; IGR1 n = 225; MeWo n = 155; SkMel13 n = 146; HEK n = 98). (**d**,**e**) Cell cycle analysis reveals S-phase accumulation in HEK and MeWo cells attributable to the overexpression of RIPK3 wt (HEK: RIPK3 KD n = 5462, RIPK3 wt n = 5307; MeWo: RIPK3 KD n = 8083, RIPK3 wt n = 24,676). The cells were fixed 48 h post-transfection using 100% ethanol for subsequent staining with propidium iodide and flow cytometry. (**a**,**c**) *t*-test for statistical analysis, ** *p* < 0.01, *** *p* < 0.001. (**b**,**d**,**e**) Fisher exact test for statistical analysis, *** *p* < 0.001.

Due to the significant involvement of RIPK3 in necroptosis, its previously reported low expression and its observed tumor suppressive effect in melanoma, we then investigated changes in cell morphology after RIPK3 overexpression and compared our results with endogenous RIPK3 induction by epigenetic editing [20]. We overexpressed RIPK3 KD (kinase dead) and RIPK3 wt (wild type) in four melanoma cell lines (Figure 3a). At 24 h post transfection, cells were fixed on glass slides. The RIPK3 KD mutant (point mutation in the kinase domain) was utilized as a negative control in comparison to wt RIPK3. RIPK3 wt and KD were fluorescence-coupled (EYFP), and the evaluation was carried out with fluorescence microscopy. The general cell morphology, the size of the cell nuclei, and their nuclear fitness were assessed. The procedure for measuring the cell nucleus size is shown in Figure S7b. In all melanoma cell lines and HEK with transfected RIPK3 wt, the cells were small and round and had less-structured nuclei and cell protrusions (Figure S8). Some cells showed cell swelling and fractured membranes (Figure S7a). In contrast, cells transfected with RIPK3 KD showed a normal cellular phenotype. The fluorescence microscopy images of RIPK3 KD showed larger cells with well-defined cell nuclei and some cell protrusions (Figure S8). The altered phenotype of the cells with RIPK3 wt was also shown by the significant deterioration in nuclear fitness vs. RIPK3 KD.

The RIPK3 wt transfected C918 cells showed a 78% reduction in normal nuclei to altered nuclei, a 57% reduction in IGR1, a 66% reduction in MeWo, a 60% reduction in SkMel13 and a 53% reduction in HEK compared to the RIPK3 KD control. In addition, after RIPK3 wt overexpression, 87% condensed, fragmented, or missing nuclei were observed in C918, 73% in IGR1, 57% in SkMel13 and 66% in HEK (Figure 3b). The categorization of the altered nuclei is shown in Figure S9. The size of the nuclei decreased significantly by using RIPK3 wt in all melanoma cell lines examined. Accordingly, the cell nucleus size in C918 was reduced by 24%, in IGR1 by 31%, in MeWo by 39%, in SkMel13 by 31% and in HEK by 37% (Figure 3c). The results show a significant deterioration of cell morphology in the cell lines C918, IGR1, MeWo, SkMel13 (all melanomas) and HEK after RIPK3 wt overexpression, which was characterized by reduced cell nuclei and reduced nuclear fitness. For the kidney cell line HEK and melanoma cell line MeWo, we performed a quantitative cell cycle analysis using flow cytometry. Cell cycle distribution of nuclei was stained by propidium iodide. In RIPK3 wt transfected cells, we detected an S-phase accumulation in comparison to the RIPK3 KD (Figure 3d,e and Figure S10).

**Figure 4:**
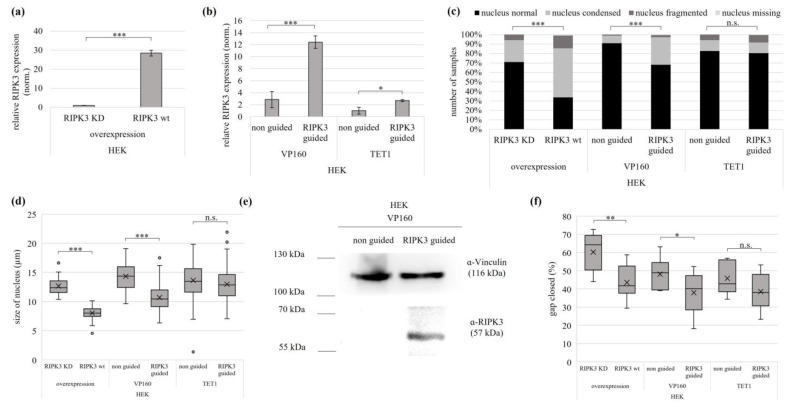
Epigenetic editing reactivates the tumor suppressive function of RIPK3 in kidney cell line. Induction by (**a**) overexpression (RIPK3 KD, RIPK3 wt in HEK cells 24 h) and by (**b**) epigenetic editing VP160-dCas9 and TET1-dCas9 (HEK, 72 and 96 h). Expression was normalized to *GAPDH* (set 1). (**c**) Induction of RIPK3 alters cell morphology. Cells after *RIPK3* overexpression were fixed on slides 24 h after transfection and 72 h/96 h (VP160, TET1) after epigenetic editing. For visualization, cells were co-transfected with EYFP and nuclei were DAPI stained (analysis by fluorescence microscopy, 64×, overexpression n = 344; VP160-dCas9 n = 352; TET1-dCas9 n = 407). Fisher exact test for statistical analysis, *** *p* < 0.001, n.s. = not significant. (**d**) Reduction in nucleus size by epigenetic editing-driven RIPK3 induction through VP160-dCas9 and TET1-dCas9. Utilizing slides prepared from (**c**), cell nucleus size was determined through fluorescence microscopy (overexpression n = 98; VP160-dCas9 331 n = 169; TET1-dCas9 n = 210). (**e**) Western blot detects RIPK3 protein after epigenetic editing induction by VP160-dCas9. HEK cells were transfected with VP160-dCas9 (72 h), lysates separated in SDS-Page and Western blotted with RIPK3 antibody (Vinculin loading control). (**f**) Reduced cell migration following RIPK3 wt overexpression and epigenetic editing induction of RIPK3 (VP160-dCas9), assessed through a wound healing assay. The scratch for the overexpression was placed 24 h post transfection, for VP160-dCas9 after 72 h, and for TET1-dCas9 after 96 h. Wound closure was determined by measuring the cell-free area at 0 h and 24 h. (**a**,**b**,**d**,**f**) *t*-test for statistical analysis, * *p* < 0.05, ** *p* < 0.01, *** *p* < 0.001, n.s. = not significant.

In further steps, we used epigenetic editing to restore endogenous RIPK3 expression and its tumor suppressive effect. For this purpose, we used the cell lines HEK and MeWo and compared the epigenetic editing results with tumor suppression of RIPK3 KD and RIPK3 wt by overexpression. To investigate the tumor suppressive effect of RIPK3 after induction by epigenetic editing, we used VP160-dCas9 and TET1-dCas9. Using quantitative expression analysis, we first demonstrated a successful RIPK3 induction by VP160-dCas9 and TET1-dCas9 in HEK (Figure 4a,b). VP160-dCas9 re-expression was 4.7-fold higher than that induced by TET1-dCas9. The VP160-dCas9-transfected HEK cells were significantly smaller and rounder. In addition, they showed hardly any protrusions and cell–cell connections, but in most cases, fit nuclei with visible nucleoli could be observed (Figure S11). In contrast, the TET1-dCas9-transfected cells showed no significant changes of RIPK3 guided compared to the non-guided control. After RIPK3 induction with VP160-dCas9, the HEK cells showed a 25% reduction in normal nuclei and a 7% reduction using TET1-dCas9 (Figure 4c). The cells showed 32% condensed, fragmented or missing nuclei after VP160-dCas9-driven RIPK3 induction and 18% using TET-dCas9. In addition, VP160-dCas9 induced RIPK3 reduced the size of the nuclei by 22%, and TET1-dCas9 still reduced the size by 5% (Figure 4d). In addition, we were also able to detect the induction RIPK3 protein using VP160-dCas9 by Western blot (Figure 4e). The detected RIPK3 protein levels correlated with the determined RIPK3 re-expression (Figure 4b).

To include further characteristics of the tumor suppressive effect of RIPK3, we investigated the proliferation/migration behavior of HEK cells after RIPK3 induction using a wound healing assay. We observed the greatest reduction in cell migration after RIPK3 wt overexpression, followed by VP160-dCas9-driven RIPK3 induction, significant for both. RIPK3 induction with TET1-dCas9 showed a reduced cell migration, which was not significant (Figure 4f and Figure S12).

**Figure 5:**
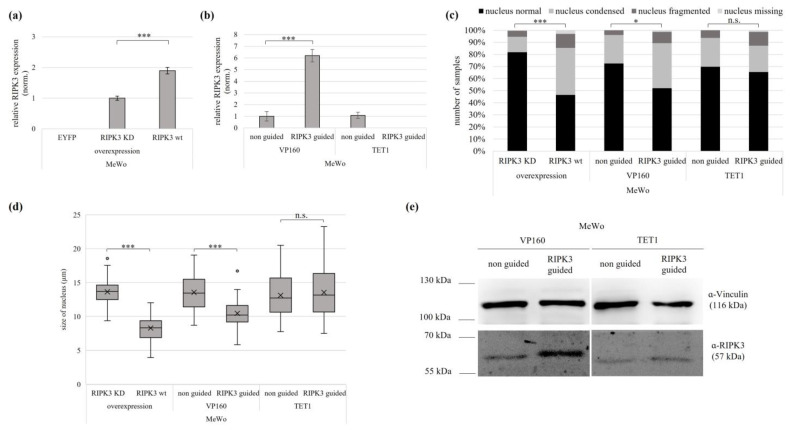
Epigenetic editing reactivates the of tumor suppressive function of RIPK3 in melanoma. Induction by (**a**) overexpression (RIPK3 KD, RIPK3 wt in MeWo cells 24 h) and by (**b**) epigenetic editing with VP160-dCas9 and TET1-dCas9 (MeWo, 96 h). Expression was normalized to *GAPDH* (set 1). (**c**) Induction of RIPK3 alters cell morphology. Cells after overexpression were fixed on slides 24 h after overexpression and 96 h after epigenetic editing. For visualization, the cells were co-transfected with EYFP, and nuclei were DAPI stained (analysis by fluorescence microscopy, 64×, overexpression n = 420; VP160-dCas9 n = 126; TET1-dCas9 n = 141). Fisher exact test for statistical analysis, * p < 0.05, *** *p* < 0.001, n.s. = not significant. (**d**) Reduction in nucleus size by overexpression and epigenetic editing. Utilizing slides prepared from (**c**), cell nucleus size was determined through fluorescence microscopy (overexpression n = 155; VP160-dCas9 n = 82; TET1-dCas9 n = 79). (**e**) Western blot detected an induced level of RIPK3 protein after epigenetic editing by VP160-dCas9. MeWo cells were transfected with epigenetic editing (96 h), lysates separated in SDS-Page and Western blotted with RIPK3 antibody (Vinculin loading control). (**a**,**b**,**d**,**e**) *t*-test for statistical analysis, * *p* < 0.05, *** *p* < 0.001, n.s. = not significant.

In melanoma MeWo cells, successful re-expression by overexpression and VP160-dCas9 was observed, but no re-expression using TET1-dCas9 (Figure 5a,b). The VP160-dCas9-transfected MeWo cells showed a reduced cellular fitness, similarly to HEK cells upon RIPK3 induction. This included smaller and rounder cells, with hardly any protrusions or cell–cell connections, but in most cases, cell nuclei were still fitter than after RIPK3 overexpression (Figure S11). After RIPK3 induction using VP160-dCas9 in MeWo, the cells showed increased numbers of altered nuclei, by 47% vs. control (condensed, fragmented or missing nuclei) (Figure 5c). Moreover VP160-dCas9-driven RIPK3 induction led to reduced nucleus size, by 23% (Figure 5d). TET1-dCas9-transfected cells showed no significant numbers of altered nuclei, compared to the control. Accordingly, RIPK3 induction by VP160-dCas9 showed deterioration in cell morphology, which was less severe compared to RIPK3 overexpression. Nevertheless, a tendency of similar effects of RIPK3 wt overexpression and RIPK3 induction by VP160 could be observed. In contrast, cells with RIPK3 induction by TET1-dCas9 showed no significant effects on their phenotype, which can be explained by the missing induction. Additionally, we observed higher levels of the RIPK3 protein using VP160-dCas9 by Western blot than in the non-guided control (Figure 4e). In summary, we were able to reestablish the tumor suppressive function of RIPK3 not only in HEK, but also in melanoma cell lines.

In conclusion, we demonstrated here the epigenetic inactivation of RIPK3 in malignant melanoma by DNA hypermethylation of its promoter region/CpG island. In addition, we showed the tumor suppressive role of RIPK3 in melanoma upon its re-induction.

## 4. Discussion

RIPK3 is an important component of necroptosis, which is a form of programmed cell death. Since the evasion of cell death is considered one of the characteristic abilities of malignant tumors, RIPK3 has already been investigated in relation to tumorigenesis [11,16]. The gene has been considered a potential tumor suppressor gene and has been linked to the development and progression of various tumor diseases [18,19,37,38]. The study by Geserick et al. studied RIPK3 in malignant melanoma and was able to show that a loss of RIPK3 results in resistance to necroptosis [20]. Findings of recent years indicate that RIPK3 is a tumor suppressor gene. However, it was unclear how the loss of RIPK3 in melanoma could be explained.

Our work, therefore, investigated the possible epigenetic regulation of RIPK3 in melanoma. Our methylation analyses of the RIPK3 CpG island, located downstream of its promoter, showed the successive hypermethylation from primary tumors to cancer cell lines, in general, but also in primary malignant melanoma. This is consistent with the concept of increasing methylation of tumor suppressor promoters in carcinogenesis [10]. We further reported that RIPK3 hypermethylation correlates with its loss of expression. Consistently, no endogenous expression could be detected in almost all cell lines, which we used for our subsequent functional analysis of RIPK3 tumor suppressive function. Our earlier studies observed the same increasing methylation pattern from naevi to primary tumors and cell lines for another tumor suppressor such as RASSF10 in malignant melanoma [24]. Interestingly, hypermethylation of RIPK3 occurs as early as in primary tumors, but is not detectable in dysplastic naevi, yet. These results highlight the great importance of early cancer detection. In larger melanoma datasets, this observation should be verified to confirm the utility of RIPK3 as an early diagnostic biomarker.

To further characterize the RIPK3 epigenetic regulation, we used a pharmacological treatment of cell lines using 5-Aza-2′deoxycytidine (Aza), an FDA- and EMA-approved medication for certain leukemias [39]. Aza is a cytidine analog and an inhibitor of DNA methyltransferases that induces passive demethylation with progressive replication [40]. We did not observe significant demethylation of *RIPK3*, despite efficient reactivation of other tumor suppressor genes using our established Aza treatment condition, in other studies also in melanoma [24,35]. In our hands, certain highly closed tumor suppressor genes always resist efficient reactivation by Aza. A higher dose of Aza cannot be recommended, due to proliferation inhibition and toxic effects. Effective Aza treatment is dependent on replication, during which Aza is incorporated into the newly synthesized DNA strand [41,42]. Dense chromatin structures (heterochromatin) are present for *RIPK3*, supported by the enrichment of EZH2 at the *RIPK3* promoter, which would hinder accessibility of the DNA. The treated cells did not grow sufficiently under Aza, and, therefore, replication-dependent Aza incorporation was not happening, and demethylation not possible. For SkMel13, we anticipate, additionally to the missing cell growth, that an Aza dose of 5 µM, which was fine for the other cell lines, is too high. In conclusion, RIPK3 resists pharmacological demethylation treatment in melanoma. Therefore, we investigated our targeted approach using epigenetic editing of *RIPK3*. Our epigenetic editing is based on a modified and optimized CRISPR/Cas9.

We used the epigenetic effectors VP160, TET1 and P300 fused to a Cas9 in our CRISPR/dCas9 system. We achieved re-expression of *RIPK3* by epigenetic editing with the effectors VP160-dCas9 and P300-dCas9 in HEK and MeWo. VP160-dCas9 induction is strong in both cell lines and must be attributed to, on the one hand, its transcriptional activation, and on the other hand, to demethylation ability in HEK. VP160-dCas9 demethylation was earlier reported by Sapozhnikov and Szyf, which we are confirming here [43]. The study by Deutschmeyer et al., which achieved similar results for the tumor suppressor gene *ZAR1*, already used a similar reactivation strategy [10]. Reactivation of *RIPK3* by P300-dCas9 was also strong in both cell lines, but no demethylation was observed. We assume that P300 directly increased acetylation of histone tails, and thereby activated the promoter by interference with the heterochromatin formation [44,45]. The effector TET1-dCas9 reactivated *RIPK3* in HEK cells, which was accompanied by a demethylation, however, not in MeWo. In the melanoma cells, possibly due to the moderate methylation of RIPK3, TET1-dCas9 did not induce its epigenetic reactivation. As a control, we used the dCas9 alone, as suggested by Sapozhnikov and Szyf, which could epigenetically reactivate promoters by steric hinderance and passive demethylation [43]. We observed in both HEK and MeWo that dCas9 alone caused an increase in *RIPK3* expression. Its increase in promoter methylation we cannot fully understand in comparison to the findings by Sapozhnikov and Szyf, but we assume an altered behavior of dCas9 without any effector in our hands [43]. Follow-up studies will clarify this issue.

In summary, we can conclude that the use of TET1-dCas9 for demethylation by epigenetic editing is dependent on a sufficiently high promoter methylation before treatment. Our observed demethylation results are due to TET1-dCas9 activity and not only due to dCas9′s steric hinderance of DNMTs, which was suggested by Sapozhnikov and Szyf, but was not observed in our hands by targeting the RIPK3 promoter [43]. The *RIPK3* promoter is epigenetically inactivated by DNA hypermethylation, and heterochromatin by EZH2 presence. Furthermore, VP160-dCas9 also proved to be the most effective modifier in our hands, so far, taking also into account our earlier study on another epigenetically inactivated gene, *ZAR1*, and confirming these earlier results [10].

Our additional experiments focused on supporting the tumor suppressor role of RIPK3 in melanoma and kidney. In HEK and in all melanoma cell lines examined, deterioration in cell morphology was observed with increased RIPK3 levels. RIPK3 expression hindered migration/proliferation and induced an Sphase accumulation during cell cycle progression. We observed that higher levels of RIPK3 due to overexpression resulted in stronger effects than moderate RIPK3 induction by epigenetic editing. However, we believe that epigenetic editing more closely mimics realistic RIPK3 levels. The functional correlation between RIPK3-induced changes of cellular morphology, migration and RIPK3-induced cell cycle arrest will be further investigated in relation to necroptosis induction. The identification of activated downstream targets of RIPK3, such as MLKL, will be investigated in further research.

For the first time, it has now become clear that RIPK3 acts as an epigenetically regulated tumor suppressor in melanoma; its re- or overexpression likely causes an induction of necroptosis, which results in an inhibition of tumor cell proliferation in melanoma and HEK. We believe that RIPK3 may serve as a cancer biomarker for melanoma in the future. In early cancer detection, the hypermethylation status of RIPK3, along with other biomarkers, could be used as a precision medicine.

## Data Availability

Although no new omics data has been generated, researchers are willing to provide additional information on protocols and samples upon request.

## Supplementary Materials

The following supporting information can be downloaded at: https://www.mdpi.com/article/10.3390/genes15020175/s1, Figure S1: *RIPK3* and its transcriptional regulators, CpG island and the guide positions; Figure S2: Increased methylation status of *RIPK3* in cancer; Figure S3: Low mutation rate of *RIPK3*; Figure S4: Increasing *RIPK3* methylation with advanced tumor stage in cancer; Figure S5: Lower expression of *RIPK3* in fibroblasts and cancer cell lines compared to normal; Figure S6: Correlating expression and methylation for *RIPK3* in melanoma within its CpG island; Figure S7: Melanoma cell membrane fractured after RIPK3 wt overexpression; Figure S8: Altered cell morphology of melanoma and kidney cell line cells after RIPK3 wt overexpression; Figure S9: Categorization of the altered nuclei after RIPK3 wt overexpression; Figure S10: Cell cycle analysis reveals S-phase accumulation in HEK and MeWo cells after RIPK3 wt overexpression; Figure S11: Reduced fitness of melanoma and kidney cells by VP160-dCas9; Figure S12: Wound healing assay shows reduced cell migration following RIPK3 wt overexpression and induction of RIPK3 using VP160-dCas9; Table S1: Summary of all analyzed samples.
