# Supplementary material for: Biomarker RIPK3 Is Silenced by Hypermethylation in Melanoma and Epigenetic Editing Reestablishes Its Tumor Suppressor Function
Source: Genes (Basel). 2024 Jan 28;15(2):175. doi: 10.3390/genes15020175 (PMC10888250; doi:10.3390/genes15020175)
Supplement: Supplementary file 1 [file genes-15-00175-s001.zip › genes-2764892-supplementary.pdf]

(a)

(b)

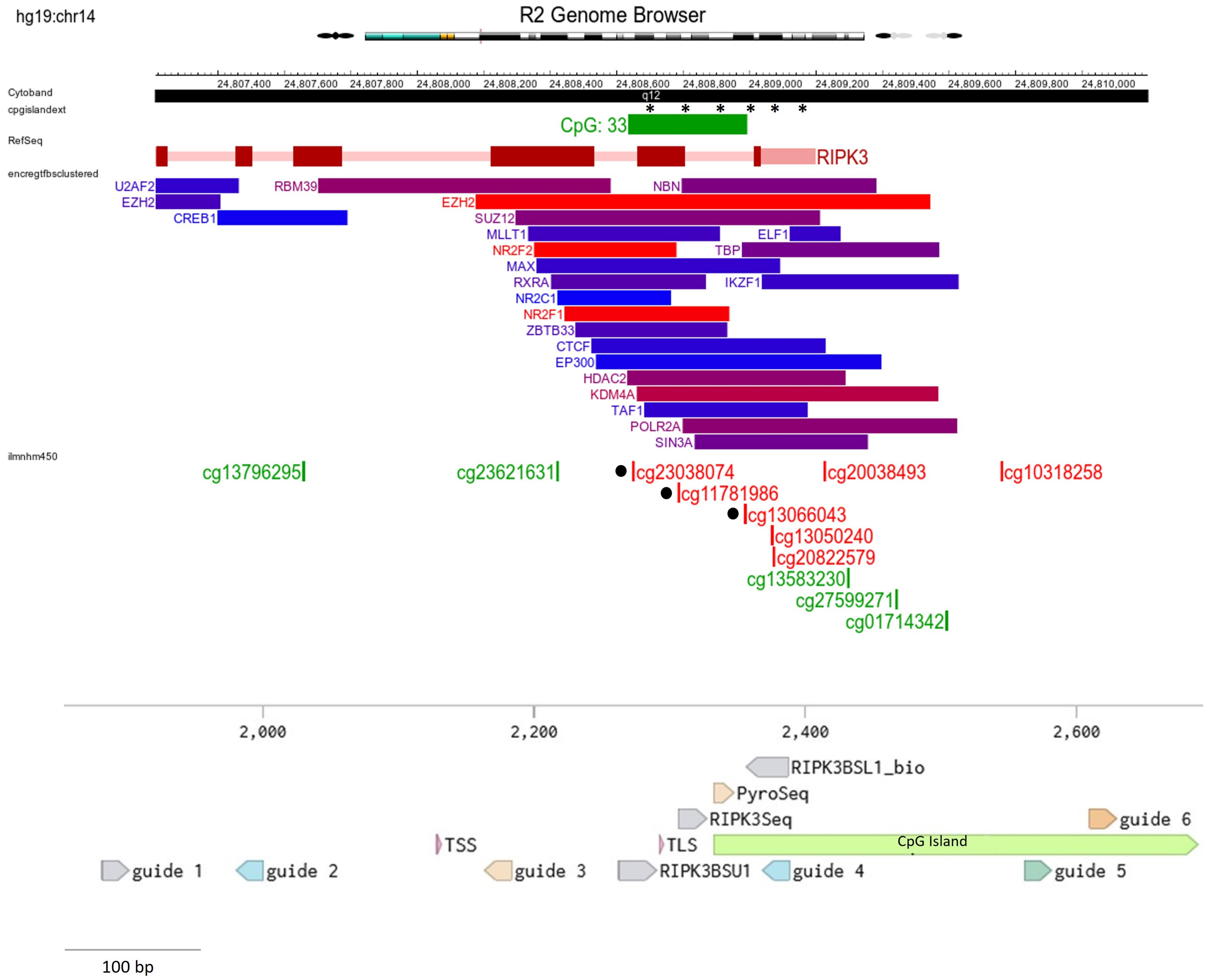

Figure S1: *RIPK3* and its transcriptional regulators, CpG island and the guide positions. **(a)** The data show the transcriptional regulators such as EZH2 binding at the promoter and CpG island region (ChIP-seq in ENCODE by R2). The CpG island is marked as a green bar. CpG probes in green are located on the upper strand, red probes on the lower strand. CpG probes marked with a dot are located in the area of the CpG island. The position of the guides is indicated with *. **(b)** Position of the guides in relation to genomic *RIPK3* structure. The guides were generated about 200 base pairs apart. The pyrosequenced area is indicated by the position of the primers (COBRA PCR: RIPK3BSU1, RIPK3BSL1_bio; Pyrosequence primer: PyroSeq). The CpG island is marked as a green bar. TSS: Transcriptional Start Site; TLS: Translation Start.

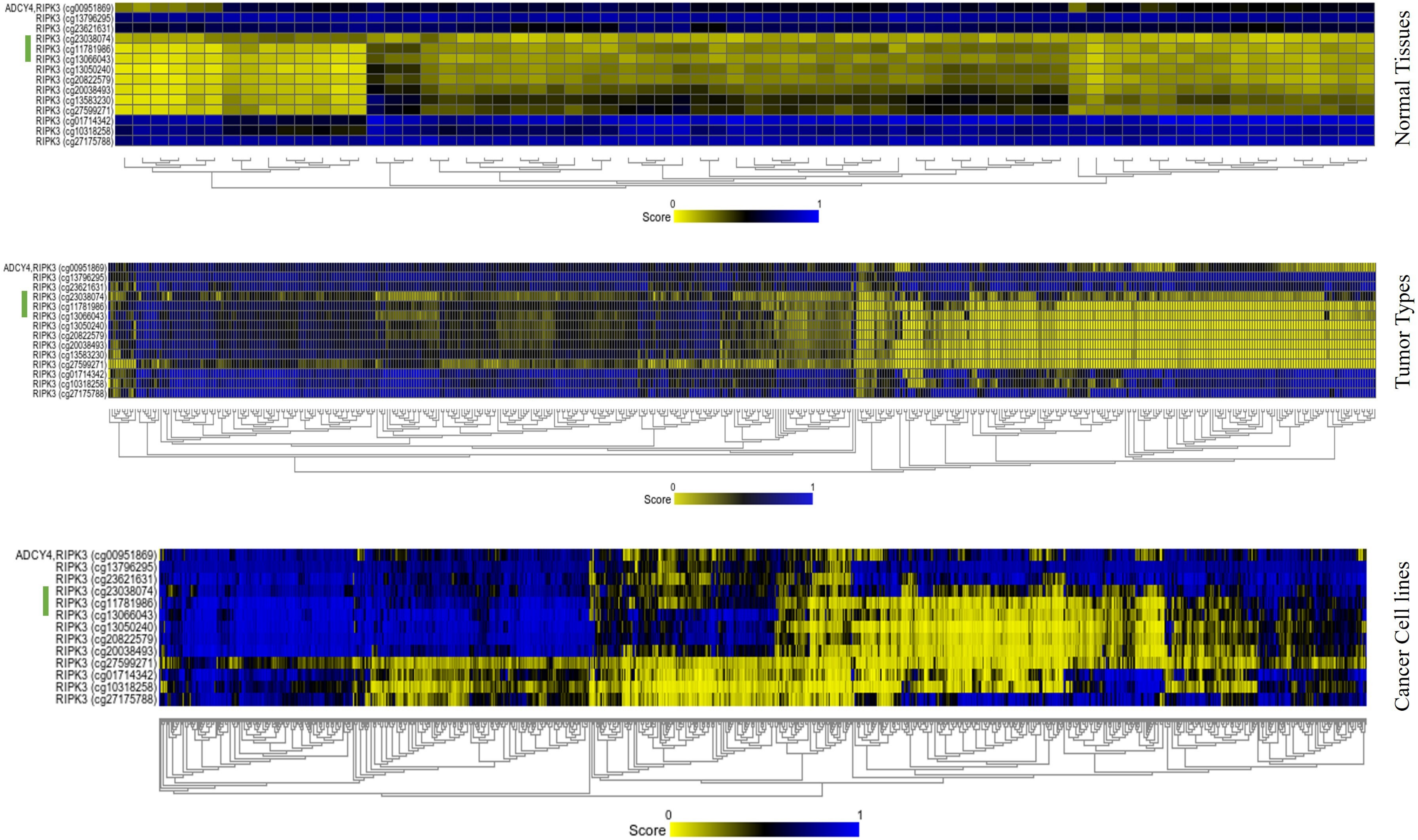

Figure S2: Increased methylation status of *RIPK3* in cancer. Increased *RIPK3* methylation in primary tumors and cell lines, compared to normael tissue, as methylation heatmaps via Illumina Methylation Assay-450k-Array (n=70, Normal Tissues-Lokk-70-custom-ilmnhm450 by R2; n=493, Tumor Types (landscape)-Heyn-493-custom-ilmnhm450 by R2; n=1028, Cell line Cancer Pharmacogenomic-Esteller-1028-custom-ilmnhm450 by R2). The CpG probes cg23038074, cg11781986 and cg13066043 of CpG island 33 of *RIPK3* are marked in green.

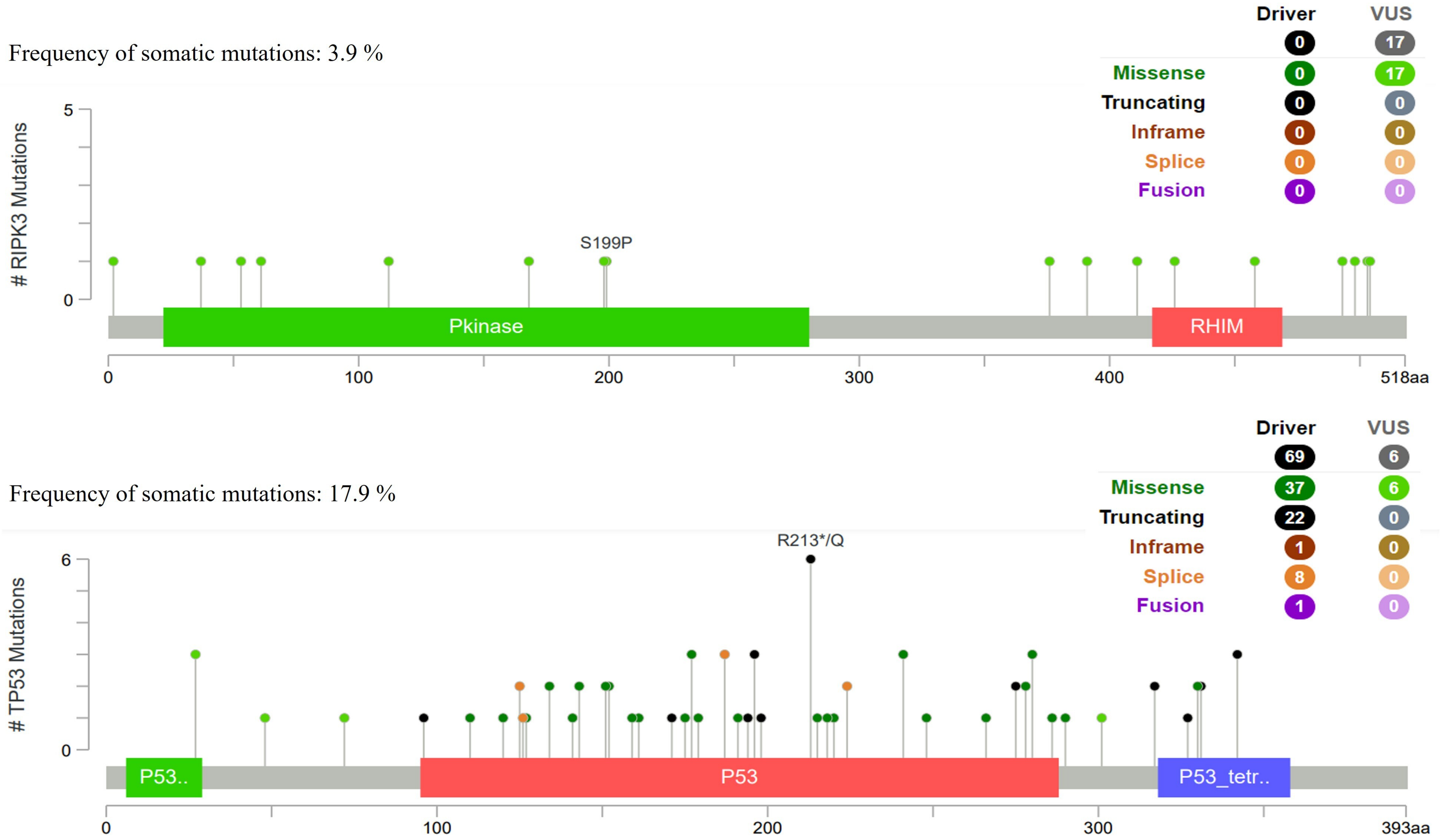

Figure S3: Low mutation rate of *RIPK3*. The proteins RIPK3 and P53 with color-coded mutations and their positions. The gene *TP53* as a example, show an increased mutational rate. The legend indicates the colors and number of driver mutations (Driver) and variant of unclear significance (VUS) mutations. aa=amino acid (TCGA, Pan Cancer Atlas by cBioPortal for Cancer Genomics).

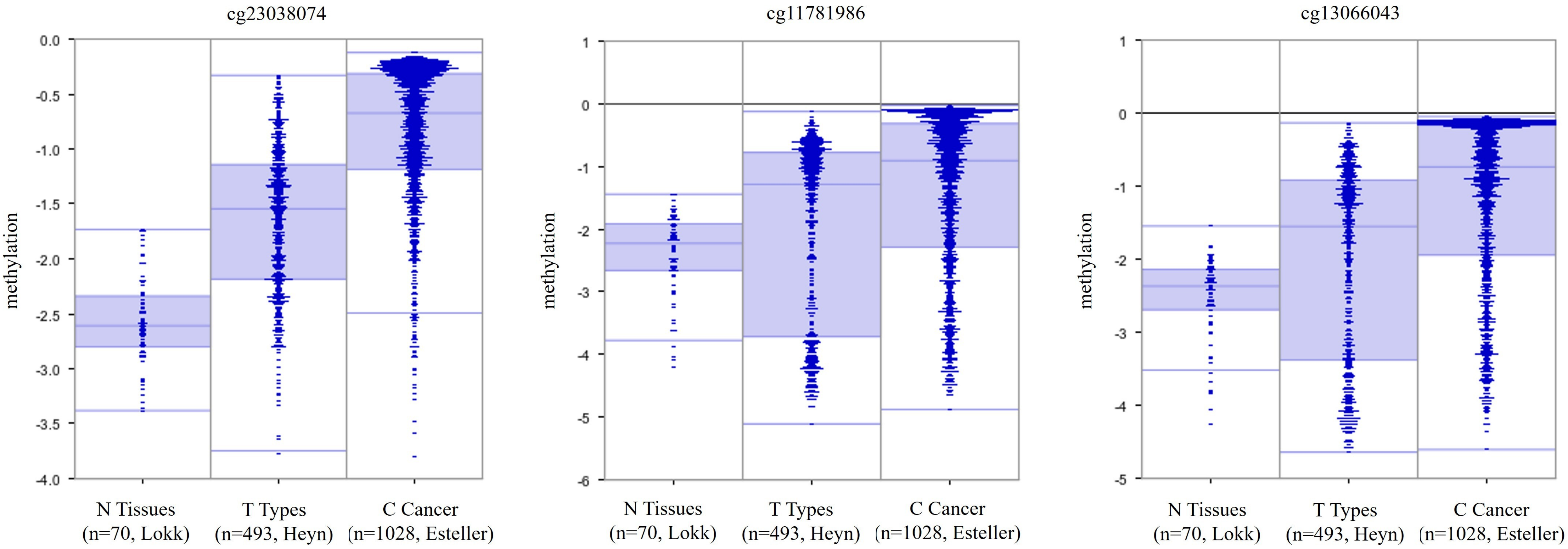

Figure S4: Increasing *RIPK3* methylation with advanced tumor stage in cancer. Hypermethylation of *RIPK3* in primary tumors and cell lines, analyzed by the three *RIPK3* CpG probes in ist CpG island (MegaSampler, n=159, custom, by R2).

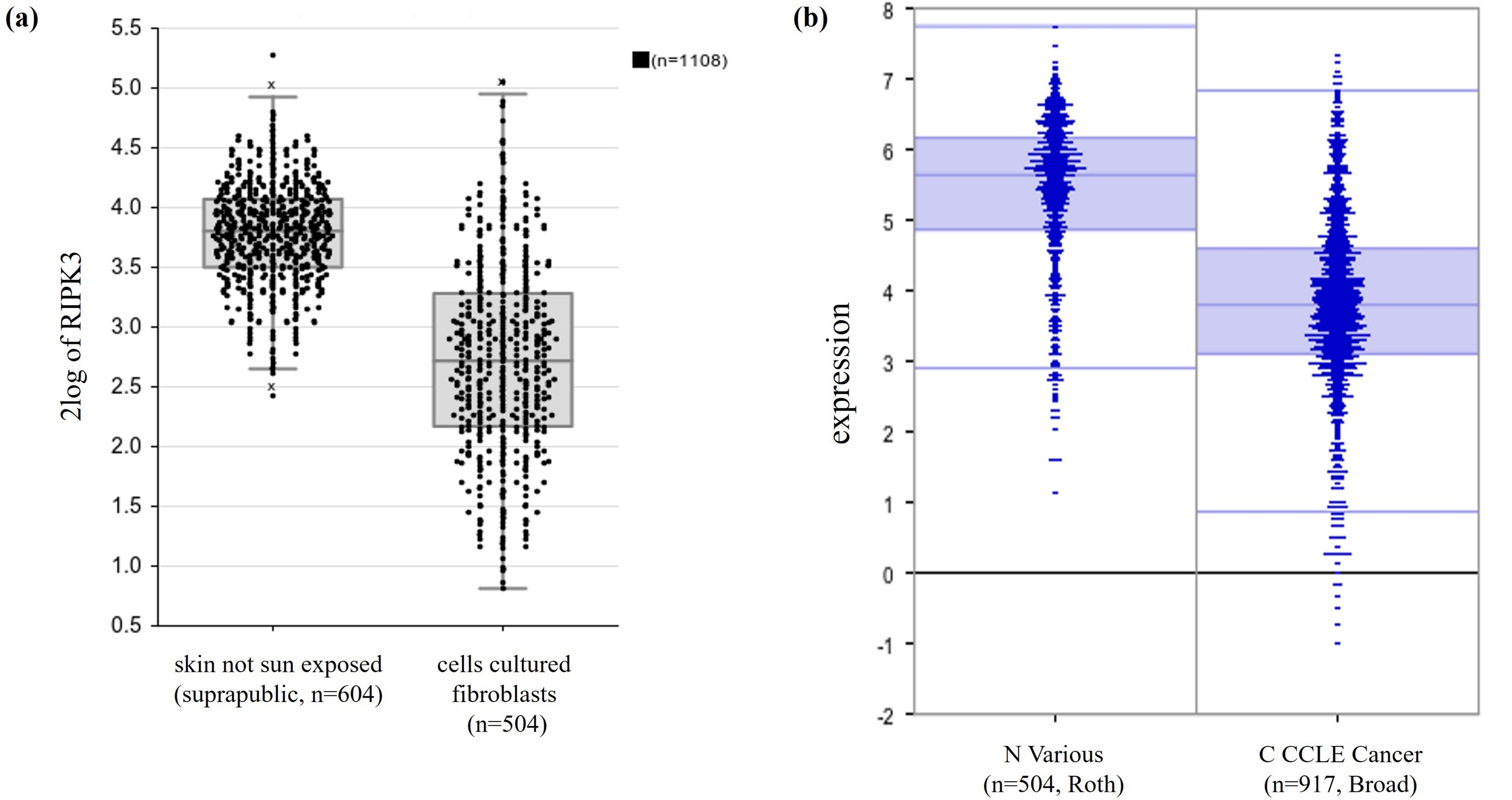

Figure S5: Lower expression of *RIPK3* in fibroblasts and cancer cell lines compared to normal. (a) Lower expression of *RIPK3* in fibroblasts from skin than in not sun exposed skin (skin not sun exposed n=604; cultured fibroblasts n=504; GTex-17382-tpm-gencode26). **(b)** Reduced expression of *RIPK3* in cancer cell lines (MegaSampler, n=1421, MAS5.0 by R2).

(a)

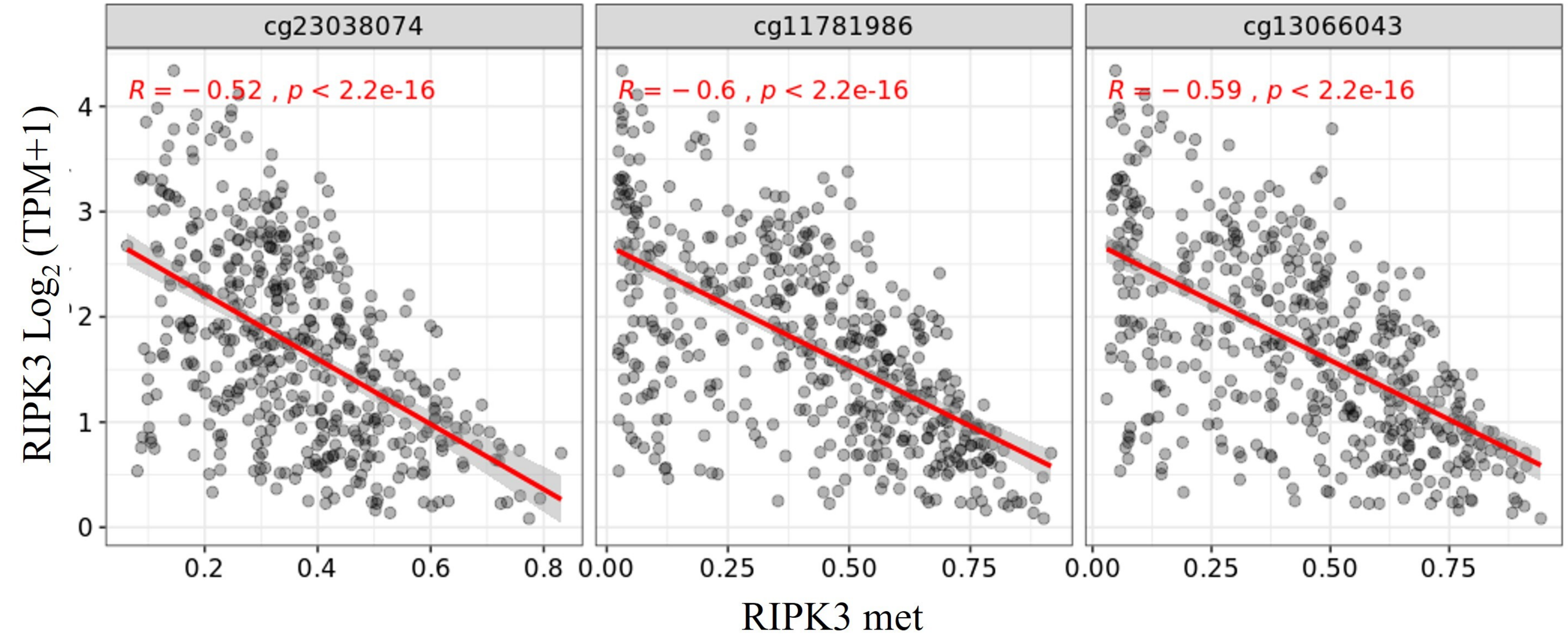

(b)

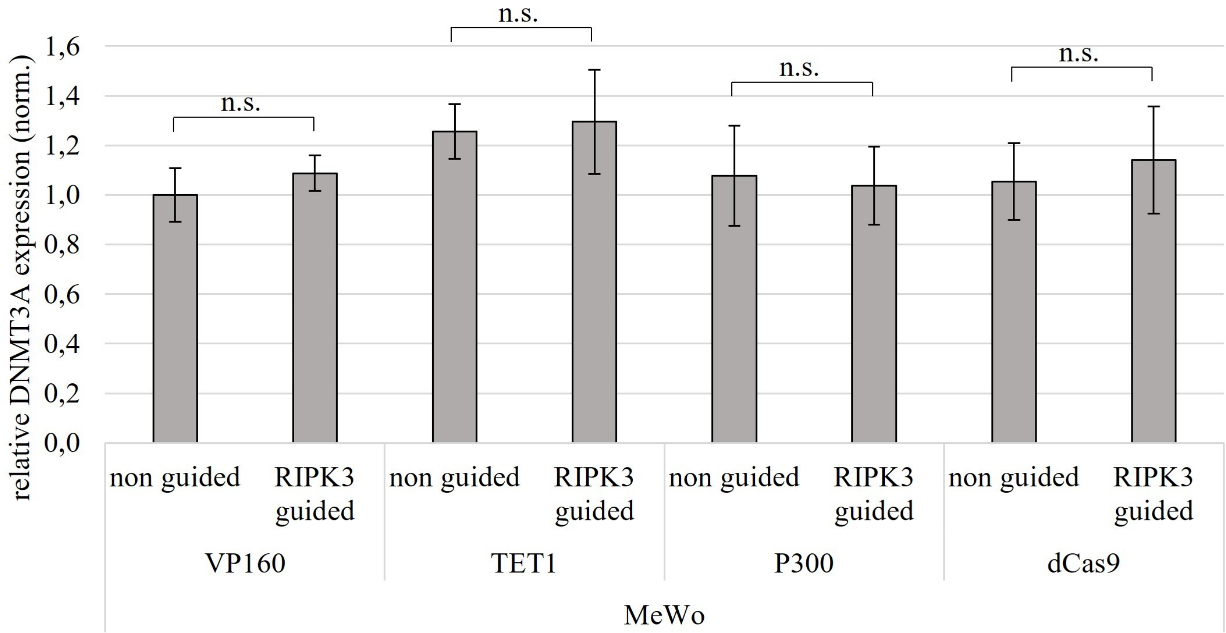

Figure S6: Correlating expression and methylation for *RIPK3* in melanoma within its CpG island. (a) The three annotated CpG probes are shown (n=470; Methylation450k, Pan-Cancer Atlas; TOIL RSEM tpm UCSC Toil RNAseq Recompute (Gene expression RNAseq) by Shiny Methylation Analysis Resource Tool). **(b)** Quantitative expression analysis in MeWo shows no difference of *DMNT3A* expression after using the different Epigenetic Editing effectors (VP160, TET1, P300, dCas9 alone). t-test for statistical analysis, *n.s.*=not significant.

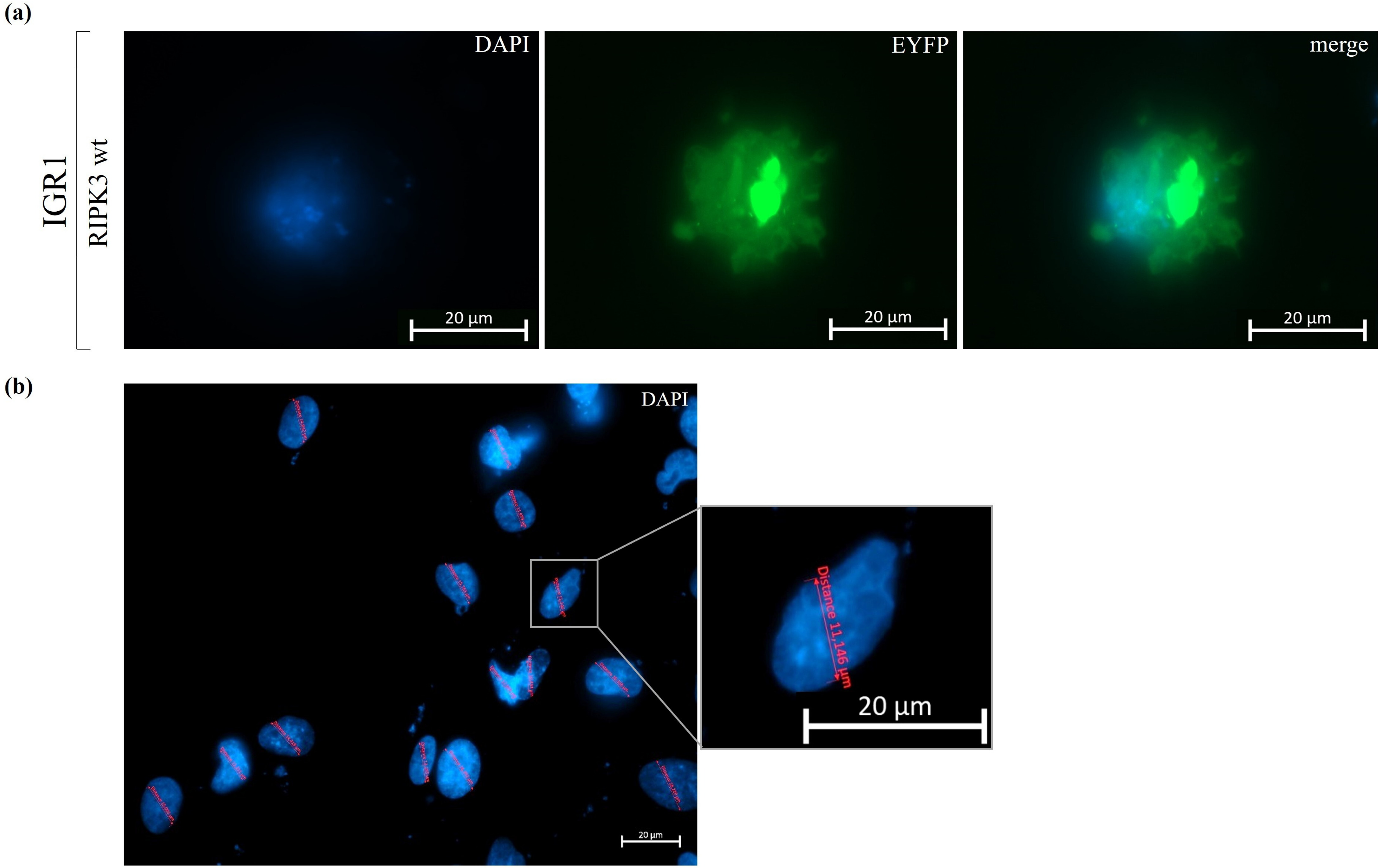

Figure S7: Melanoma cell membrane fractured after RIPK3 wt overexpression. (a) IGR1 melanoma cells were transfected with pEYFPC2-RIPK3 wt and fixed on slides 24 hours post transfection. The slides were analyzed by fluorescence microscopy (64x objective). Nuclear staining was performed by DAPI. **(b)** Exemplary representation of the cell nucleus measurement in SkMel13 with RIPK3 KD. The diagram illustrates the procedure for measuring the cell nuclei sizes, which were determined as part of the phenotypic determination. The evaluation was carried out using fluorescence microscopy. The cell nuclei were stained with DAPI.

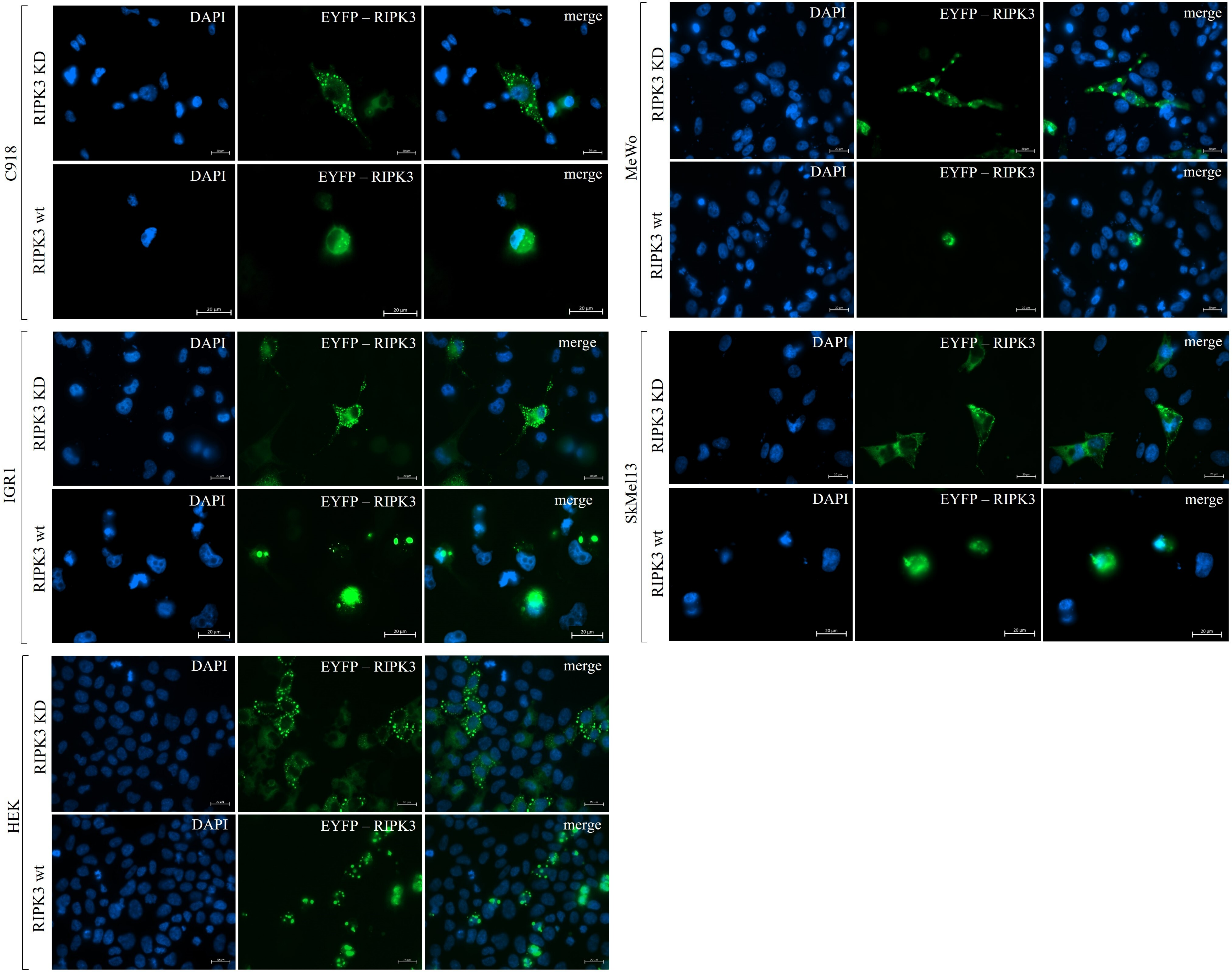

Figure S8: Altered cell morphology of melanoma and kidney cell line cells after RIPK3 wt overexpression. Cells were transfected with pEYFPC2-RIPK3 wt and fixed on slides 24 hours post transfection. As negative control pEYFP-C2 RIPK3 KD was used. The slides were analyzed by fluorescence microscopy (64x objective). Nuclear staining was performed by DAPI.

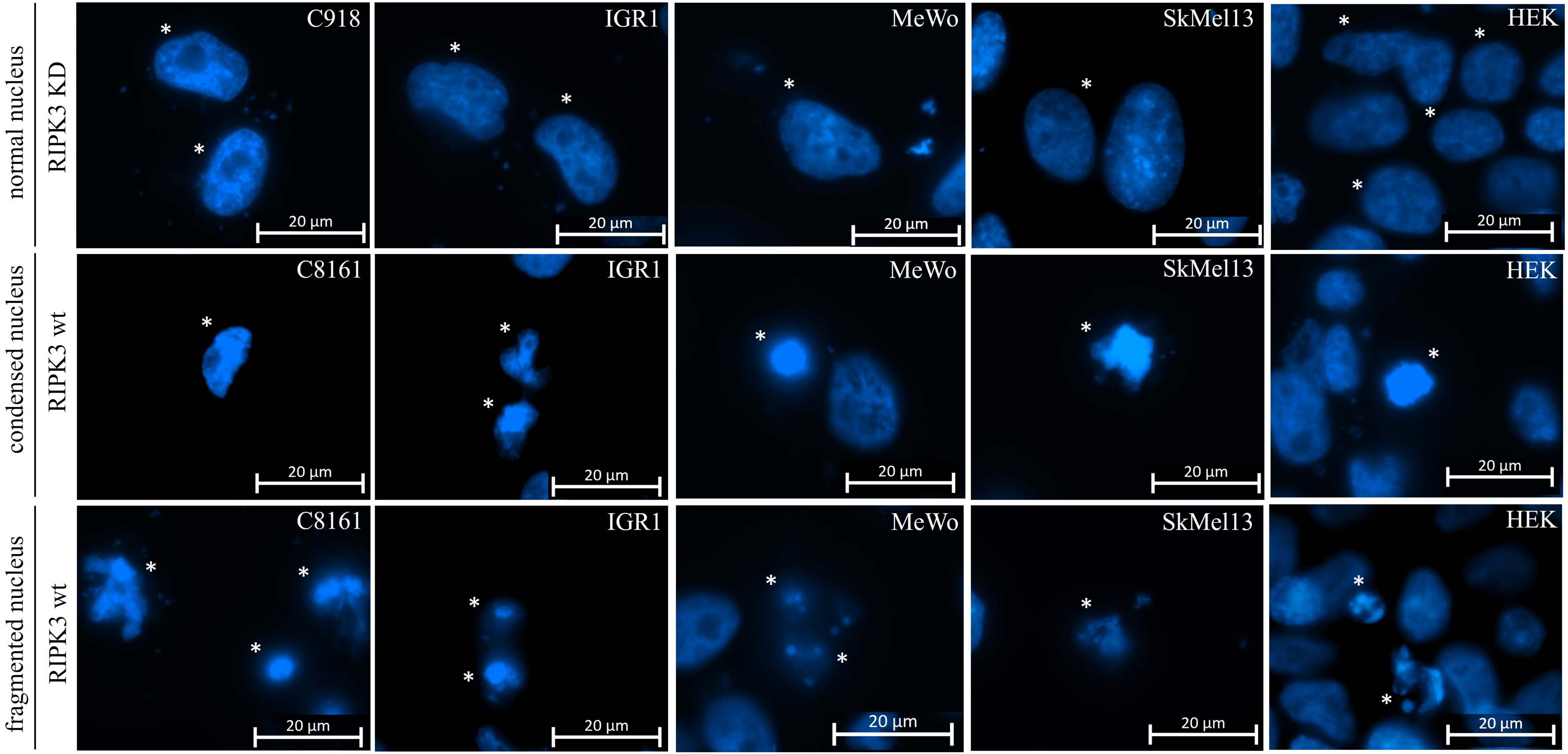

Figure S9: Categorization of the altered nuclei after RIPK3 wt overexpression. Cells were transfected with pEYFPC2-RIPK3 wt and fixed on slides 24 hours post transfection. The slides were analyzed by fluorescence microscopy (64x objective). Nuclear staining was performed by DAPI. * marks transfected cells and their according nuclei.

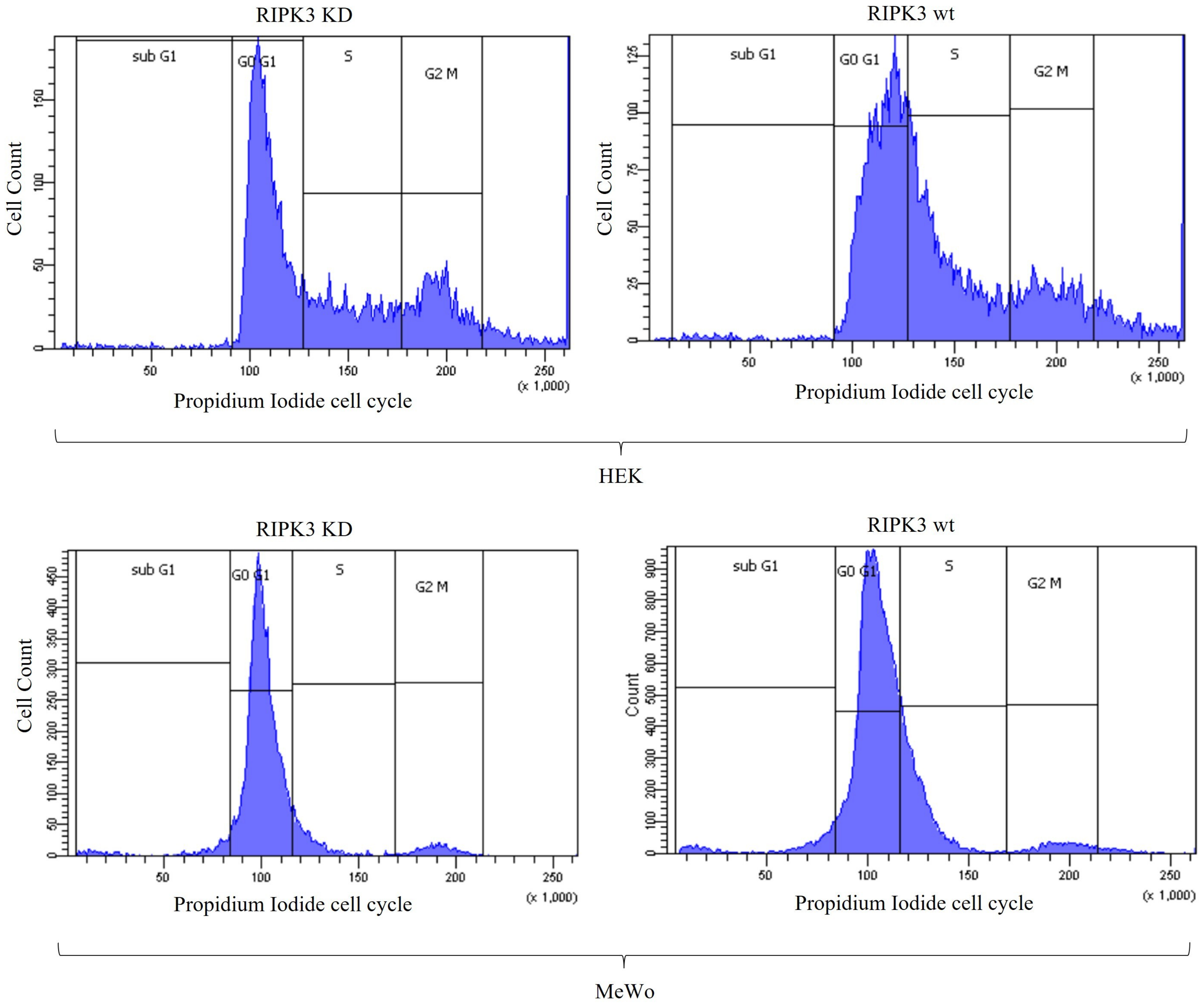

Figure S10: Cell cycle analysis reveals S-phase accumulation in HEK and MeWo cells after RIPK3 wt overexpression. HEK: RIPK3 KD n=5462, RIPK3 wt n=5307; MeWo: RIPK3 KD n=8083, RIPK3 wt n=24676. The cells were fixed 48 hours post-transfection using 100% ethanol for subsequent staining with Propidium Iodide and analysis by flow cytometry.

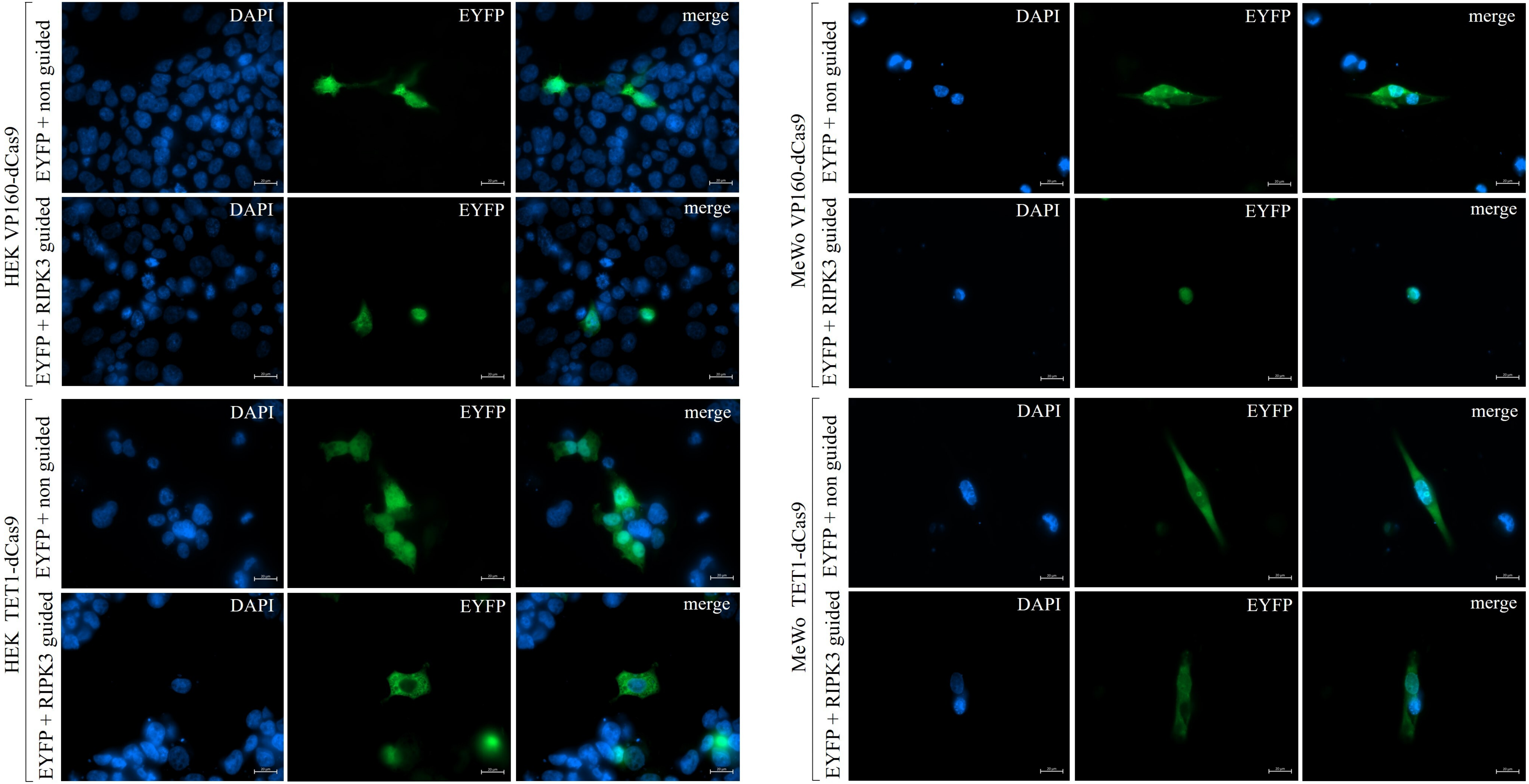

Figure S11: Reduced fitness of melanoma and kidney cells by VP160-dCas9. The Epigenetic Editing transfected cells were fixed on slides after 96 hours. For visualization, we co-transfected VP160-dCas9 and TET1-dCas9 with EYFP (1:8) respectively. The slides were analyzed by fluorescence microscopy (64x objective). DAPI was used for nuclear staining.

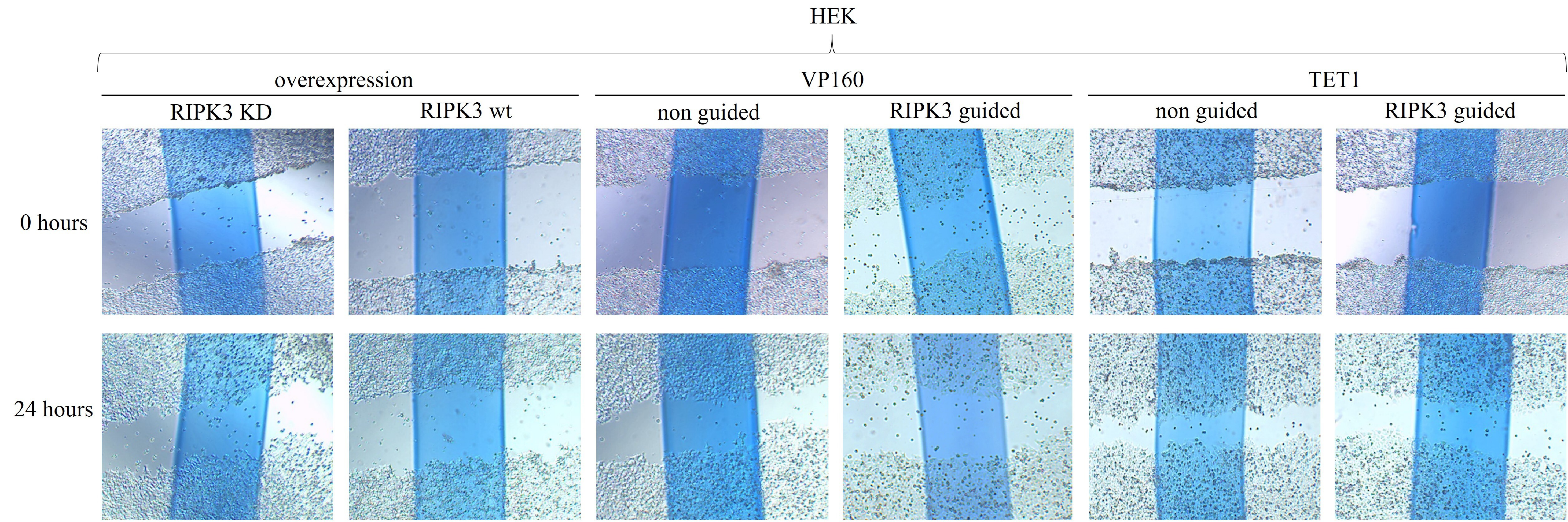

Figure S12: Wound healing assay shows reduced cell migration following RIPK3 wt overexpression and induction of RIPK3 using VP160-dCas9. The scratch for the overexpression was initiated 24 hours post transfection, for VP160-dCas9 after 72 hours, and for TET1-dCas9 after 96 hours. Wound closure was determined by measuring the cell free area at 0 h and 24 h. Representative pictures to Figure 4 (f).

Table S1: Summary of all analyzed samples.

Tissue	Type	Name	Gender	Age	Source	Pyrosequencing (mean methylation of RIPK3)
Skin	Cell line (melanoma)	C918	F	60	CVCL_8471	100,00
		IGR1	M	42	CVCL_1303	48,33
		MeWo	M	78	CVCL_0445	35,67
		SkMel13	M	65	CVCL_6022	69,67
		SkMel19	M	50	CVCL_6025	90,33
Skin	Aza-treated Cell line (5 µM) (melanoma)	C918	F	60	CVCL_8471	89,33
		IGR1	M	42	CVCL_1303	45,00
		MeWo	M	78	CVCL_0445	43,00
		SkMel13	M	65	CVCL_6022	85,33
		SkMel19	M	50	CVCL_6025	91,67
Skin	Primary sample nevus cell nevus	NZN8	F	16	Dermatology Heidelberg	8,00
		NZN15	F	40		3,67
		NZN16	M	42		15,00
		NZN37	F	18		4,00
		NZN42	F	40		7,00
		NZN46	F	21		7,00
Skin	Primary sample dysplastic nevus cell nevus	NZN2	M	55	Dermatology Heidelberg	2,67
		NZN21	M	25		3,33
		NZN23	M	39		6,00
		NZN33	M	56		3,33
		NZN34	F	45		5,67
		NZN54	M	38		2,33
		NZN57	M	34		11,33
Skin	Primary sample primary melanoma	MM40	M	73	Dermatology Heidelberg	7,00
		MM41	M	70		87,00
		MM44	M	75		3,33
		MM61	M	65		25,67
		MM65	F	76		17,00
		MM67	F	92		96,67
		MM68	F	92		18,00
		MM69	F	92		42,00
		MM70	F	92		4,00
		MM72	F	92		8,00
		MM74	F	92		10,33
		MM100	F	50		13,33
		MM101	N/A	N/A		9,33
		MM104	N/A	N/A		7,00
		MM107	N/A	N/A		7,00
		MM109	F	82		4,67
		MM127	M	68		5,33
		MM128	M	62		2,67
		MM129	M	65		9,67
		MM130	M	46		3,67
		MM131	M	55		11,00
		MM137	M	75		6,00
		MM138	M	62		8,33
		MM139	M	60		8,33
Skin	Primary sample metastases lymph node	MM59a	N/A	N/A	N/A	44,33
		MM159	N/A	N/A		82,00
Skin	Primary sample metastases brain	NP1	N/A	N/A	N/A	61,00
		NP5	N/A	N/A		70,33
		NP6	N/A	N/A		99,50
		NP7	N/A	N/A		61,00
		NP8	N/A	N/A		20,67
		NP18	N/A	N/A		49,67
		NP23	N/A	N/A		20,33
Skin	Primary sample metastases skin	MM78	F	92	Dermatology Heidelberg	57,67
		MM80	F	56		75,33
		MM81	F	56		10,33
		MM83	F	56		36,67
		MM86	N/A	N/A		50,67
		MM87	M	81		44,67
		MM88	M	81		51,00
		MM91	F	60		56,67
		MM92	F	60		72,00
		MM97	M	79		69,67

further information available on request, Heidelberg Germany
